# Chymase Activity in Plasma and Urine Extracellular Vesicles in Primary Hypertension

**DOI:** 10.34067/KID.0000000000000555

**Published:** 2024-08-22

**Authors:** Sarfaraz Ahmad, Gagan Deep, Henry A. Punzi, Yixin Su, Sangeeta Singh, Ashish Kumar, Shalini Mishra, Amit K. Saha, Kendra N. Wright, Jessica L. VonCannon, Louis J. Dell’Italia, Wayne J. Meredith, Carlos M. Ferrario

**Affiliations:** 1 Laboratory of Translational Hypertension, Department of Surgery, Wake Forest School of Medicine, Winston Salem, North Carolina; 2 Department of Internal Medicine, Wake Forest School of Medicine, Winston Salem, North Carolina; 3 J Paul Sticht Center for Healthy Aging and Alzheimer's Prevention, Wake Forest School of Medicine, Winston-Salem, North Carolina; 4 Wake Forest Baptist Comprehensive Cancer Center, Wake Forest School of Medicine, Winston-Salem, North Carolina; 5 Punzi Medical Center, Carrollton, Texas; 7 Department of Anesthesiology, Wake Forest School of Medicine, Winston Salem, North Carolina; 8 Division of Cardiovascular Disease, Department of Medicine, The University of Alabama at Birmingham, Birmingham, Alabama; 9 Department of Veterans Affairs, Birmingham Veterans Affairs Health Care System, Birmingham, Alabama; 6 UT Southwestern Medical Center, Dallas, Texas

**Keywords:** angiotensin, biomarkers, cell signaling, CKD, endocytosis, endothelial cells, hypertension, MRNA, renal ischemia, renin angiotensin system

## Abstract

**Key Points:**

- Blood and urine extracellular vesicles isolated from hypertensive patients possess high chymase enzymatic activity.
- Chymase activity was significantly higher in small extracellular vesicles obtained from hypertensive patients with suboptimal BP control.

**Background:**

Circulating extracellular vesicles (EVs) carry protected cargoes of nucleic acids, proteins, and metabolites. In this study, we identified and validated the surface proteins and enzymatic activity of chymase, angiotensin converting enzymes 1 (ACE) and 2 (ACE2), and neprilysin (NEP) in EVs isolated from the blood and urine of primary hypertensive patients.

**Methods:**

Peripheral venous blood and spot urine from 34 hypertensive patients were processed to isolate plasma and urinary EVs. Immunogold labeling and transmission electron microscopy validated the presence of the exosomal marker protein CD63 on the surface of plasma and urinary EVs. Flow cytometry characterized plasma and urinary EVs for CD63, CD9, and CD81 surface markers. In addition, exosomal CD63, TSG101, and Alix were analyzed in urine by western blotting. Urinary EVs did not express the endoplasmic reticulum protein calnexin and Golgi protein GM130. Chymase, ACE, ACE2, and NEP activities on ^125^I substrates—angiotensin-(1–12) (Ang-[1–12]) and angiotensin II—(1 nmol/L each) were quantified by HPLC. Data were analyzed based on whether the patient's BP was controlled (group 1: <140/80 mm Hg) or noncontrolled (group 2: ≥140/80 mm Hg).

**Results:**

Chymase activity on Ang-(1–12) was significantly higher in plasma and urinary EVs than in ACE, ACE2, and NEP. In addition, chymase activity in urine EVs was more than three-fold higher than in plasma EVs. Chymase activity increased in plasma and urine EVs retrieved from group 2 patients. No comparable differences were found in the enzymatic activities of ACE, ACE2, and NEP urinary EVs between group 1 and group 2.

**Conclusions:**

These studies reveal a differential enzymatic activity of renin angiotensin system enzymes in plasma and urine EVs isolated from hypertensive patients. Demonstrating a comparatively high chymase enzymatic activity in EVs expands a previously documented finding of increased plasma Ang-(1–12) in hypertensive patients.

## Visual Abstract

**Figure:**
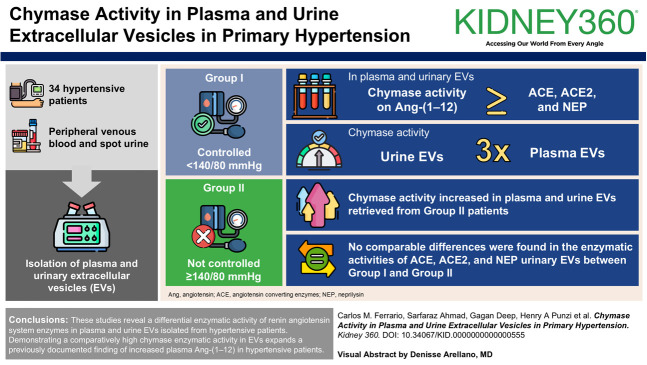

## Introduction

The 2023 report on high BP by the World Health Organization^1^ reveals a doubling of people living with hypertension (from 650 million to 1.3 billion between 1990 and 2019).^2^ Given the overlap of hypertension with type 2 diabetes, obesity, and CKD, a systematic effort to unravel the bidirectional interactions between hypertension and cardio-renal-metabolic syndrome would reduce the burden of these interconnected chronic diseases.

Extracellular vesicles (EVs) have gained credence from the demonstration that circulating microvesicles predict poor clinical outcomes.^3,4^ Endosomal-derived EVs (<200 nm) transport a variety of bioactive molecules.^5^ We posited that EVs released from cardiovascular tissues could become biomarkers of renin angiotensin system (RAS) activity, oxidative stress, and vascular inflammation.^6,7^

Compensatory activation of alternate enzymatic pathways for angiotensin II (Ang II)-induced pathology after RAS pharmacotherapy contributes to suboptimal management of human hypertension.^8,9^ The identification of angiotensin-(1–12) (Ang-[1–12]) as an alternate tissue forming Ang II substrate by chymase reveals a contribution of non-renin and non-ACE mechanisms to the pathophysiology of primary hypertension.^10–12^ The demonstration of high plasma Ang-(1–12) levels in primary hypertensive patients, naïve to antihypertensive therapy^13^ or expressing resistant hypertension,^9,14^ is in keeping with this hypothesis.

This study characterized for the first time the main enzymatic activities of chymase, angiotensin converting enzymes 1 and 2 (ACE and ACE2, respectively), and neprilysin (NEP), ferried by EVs isolated from the plasma and urine of hypertensive patients.

## Methods

Thirty-four patients (age range, 31–80 years; five female) attending an outpatient clinic at the Trinity Hypertension Research Institute (Carrollton, TX) consented to participate in the study. The research was conducted according to the guidelines of good clinical practice, approved by the Sterling Institutional Review Board (Sterling, Atlanta, GA, Institutional Review Board 7175-HAPunzi), and followed the ethical principles outlined in 2023 Declaration of Helsinki.^15^ The consenting procedure included assessing competence to read, comprehend, and sign the consent form before participating in any study procedures.

A medical history, including medications, was taken during the initial visit. Drug compliance was verified *via* prescription refills because all of these patients were recruited from the active patient registry at the Trinity Hypertension Research Institute. Patients included in this study had been seen every 3 months before starting the study. Demographic information (age, sex, and ethnicity) and clinical characteristics were recorded in all patients (Table 1). Triplicate measures of seating systolic BP (SBP) and diastolic BP were obtained with a mercury sphygmomanometer on both arms in patients instructed to avoid ingestion of caffeine-containing beverages, cigarette smoking, or physical activity for at least 30 minutes. Pulse rate was obtained at the 2- to 5-minute interval between the second and third seated BP readings. An antecubital vein blood sample (7 ml) was collected in tubes containing an inhibitor cocktail, as described by us previously.^16^ A 20 ml spot urine sample was obtained from all 34 patients. The urine, collected in sterile cups containing 1 N hydrochloric acid,^17^ was centrifuged at 3000 g for 10 minutes. Plasma (1.0 ml) and urine (5.0 ml) aliquots were stored at −80°C.

**Table 1:** Demographics and clinical characteristics

Variable	Values	
	Mean±SD	Range
Age, yr	63±11	31–80
Sex, male/female	29/5	
Body weight, kg	97±24	64–179
**Group I. Controlled hypertensives (*n*=24)**		
SBP, mm Hg	128±9	110–139
DBP, mm Hg	82±9	69–99
Mean arterial pressure, mm Hg	97±8	84–112
Pulse pressure, mm Hg	47±8	32–59
Heart rate, beats/min	71±8	60–86
Body mass index, kg/m^2^	33±6	25–51
**Group II. Noncontrolled hypertensives (*n*=10)**		
SBP, mm Hg	147±8^a^	140–167
Diastolic BP, mm Hg	91±9^a^	80–110
Mean arterial pressure, mm Hg	110±8^b^	100–129
Pulse pressure, mm Hg	56±6	50–69
Heart rate, beats/min	74±9	60–84
Body mass index, kg/m^2^	30±5	25–39

DBP, diastolic BP; SBP, systolic BP.

a *P* < 0.0001 versus group 1.

b *P* < 0.0002 versus group 1.

Antihypertensive medications used by these patients included ACE inhibitors (four patients), beta-blockers (ten patients), calcium channel blockers (14 patients), diuretics (14 patients), and Ang II receptor blockers (21 patients). Twenty patients were on combination therapy, nine were on monotherapy, and five were untreated. Drug compliance was verified *via* prescription refills. Of the 34 patients enrolled in the study, 24 were defined as having their hypertension controlled (<140/90 mm Hg) while ten others were defined as noncontrolled (≥140/90 mm Hg) at the time of entering the study.

### EV Isolation

Plasma EVs were isolated by a modified immunoprecipitation method using ExoQuickTM (EXOQ20A-1, System Biosciences, CA) and from urine by ultracentrifugation.^18,19^ Details of the procedures are incorporated in the Supplemental Material.

### Immunogold Labeling and Transmission Electron Microscopy

EVs were visualized using transmission electron microscopy after immunogold labeling.^19^ EVs were fixed with 4% paraformaldehyde for 10 minutes at room temperature and adsorbed on 200 mesh Copper grids (with carbon-coated formvar film) activated with 100% ethanol. Additional details of the procedures are described in the Supplement Material.

### Flow Cytometry

Flow cytometry characterized the percentage of typical tetraspanin markers (CD63, CD9, and CD81), as described previously.^19,20^ EVs were labeled with membrane labeling dye CellBrite 488 (Biotium, CA) with or without the CD63-PE (353004) and CD81-PE (349506) (BioLegend, CA) and CD9-APC (Invitrogen: 17-0091-82) antibodies. EVs without dye were used as a control to set the gate for positively labeled EVs. EVs labeled with dye but without CD63-PE/CD81-PE/CD9-APC antibodies were used to set the gate for APC/PE-positive events. CD63-PE, CD81-PE, and CD9-APC antibodies and dye were also analyzed at the same dilution in PBS (filtered through a 0.1-*μ*m filter). All samples were acquired on CytoFlex (Beckman Coulter Life Science, IN) for 60 seconds at a low flow rate. Filtered PBS was run between the samples.

### Nanoparticle Tracking Analysis

Hydrodynamic size and concentration of EVs were analyzed using nanoparticle tracking analysis (Nanosight NS300, Malvern Instruments, United Kingdom), equipped with violet laser (405 nm).^19^ The instrument was prepared using PBS (pH 7.4) at 25°C. The average of 5–30 seconds videos from every sample was recorded as the final size and concentration of plasma and urine EVs.

### Western Blotting

Twenty-five microgram of urine EVs were separated on 12% SDS page gel and transferred to a nitrocellulose membrane. Blots were blocked with 5% nonfat milk for 1 hour and incubated with anti-CD63 (1:500; PA5-92370, Invitrogen, Thermo Fisher Scientific Inc.), TSG101 (1:500; ab83, Abcam), Alix (1:500, ab88388, Abcam, Cambridge, United Kingdom), calnexin (1:500, ab22595, Abcam), and GM130 (1:500, NBP2-53420, Novus Biologicals, Centennial, CO) antibody overnight at 4°C. Membranes were washed with 0.1% phosphate buffered saline with tween and incubated with appropriate secondary antibody for 120 at room temperature. Blots were developed with enhanced chemiluminescence reagent (Bio-Rad Inc., CA). In some instances, membranes were stripped and reprobed for another protein of interest.

### RAS Enzyme Activity Assays

RAS enzyme activities (chymase, ACE, ACE2, and NEP) were measured in isolated EVs by HPLC using highly purified radiolabeled substrates as described by us.^17^ Additional details of the method are incorporated in the Supplement Material. Enzyme activities were calculated based on the amount of parent ^125^I-Ang substrate hydrolyzed into specific ^125^I-Ang products by the EVs in the absence and the presence of specific inhibitors. All enzyme activities are reported as fmol×min^−1^×mg^−1^.

### Angiotensin Peptides Radiolabeling and Purification

Human Ang-(1–12) (DRVYIHPFHLVI) and Ang II (DRVYIHPF) were radiolabeled with ^125^Iodine-[^125^I] at the tyrosine fourth residue [Y] using oxidant chloramine-T and purified in a C18 column by HPLC.^14^ Details of the assay procedures are incorporated in the Supplemental Material.

### Statistical Analysis

Statistical analysis was performed using IBM SPSS Statistics version 26.0 (IBM Corporation, NY) and GraphPad PRISM software (San Diego, CA). Normal data distribution was evaluated using the Shapiro–Wilk Normality and Kolmogorov–Smirnov goodness-of-fit tests. Data were log transformed because enzyme activity values failed the Shapiro–Wilk Normality test. We used the Pearson correlation test to determine relationships between RAS enzyme activities and other variables. We tested differences in values for log-transformed RAS enzyme activities using a mixed-mode ANOVA. Differences between groups were evaluated with the Student *t* test. *P* < 0.05 was considered statistically significant.

## Results

Twenty nine of 34 patients were male; 22 reported being White, 9 declared to be Hispanics, two were Black, and one was Asian. Although 85% of patients (29 of 34) were using antihypertensive medications, a SBP <140/80 mm Hg was present in 24 patients (group 1, Table 1) and above (≥140/80 mm Hg) in ten others (group 2, Table 1). Differences in risk factors or classes of antihypertensive agents influenced whether patients were included in group 1 (controlled hypertensives) or group 2 (noncontrolled hypertensives).

**Figure 1:**
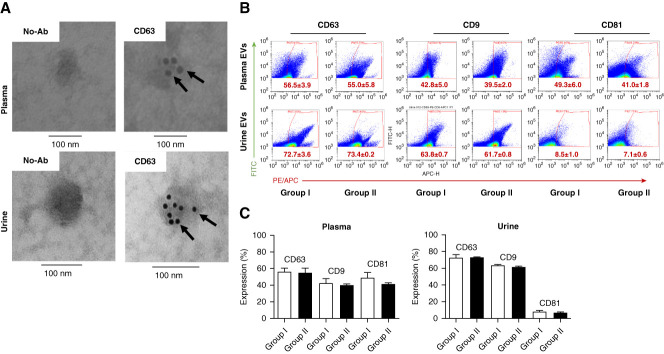
**TEM and flow cytometry validate plasma and urinary EVs.** The isolated plasma and urinary EVs were detected with rabbit anti-CD63 primary antibody and immunogold labeled anti-rabbit IgG secondary antibody by TEM (A) and characterized for the EVs surface markers for CD63, CD9, and CD81 by flow cytometry (B), as described in the Methods. The flow cytometry data ([C] scatter plots and bar graphs; mean±SEM) showing the expression of CD63, CD9, and CD81 on plasma and urine EVs (*n*=3 in each group). EV, extracellular vesicle; No-Ab, no anti-CD63 primary antibody present; PE/APC, phycoerythrin /allophycocyanin; TEM, transmission electron microscopy.

EVs extracted from hypertensive patients were characterized according to the criterion outlined for Minimal Information for Studies of Extracellular Vesicles, 2018 guidelines.^22^ The presence of the exosomal marker protein CD63 on the surface of EVs was validated by immunogold labeling transmission electron microscopy (Figure 1A).^18–20^ Plasma and urine EVs from group 1 (controlled hypertensives) and group 2 (noncontrolled hypertensives) patients were further characterized by flow cytometry for surface markers (CD63, CD9, and CD81) (Figure 1B). No differences in expression of surface markers CD63, CD9, and CD81 were detected in the plasma and urine of groups 1 and 2 (Figure 1C). However, the surface marker CD81 expression on urine EVs was notably lower than that of plasma EVs (Figure 1C).

**Figure 2:**
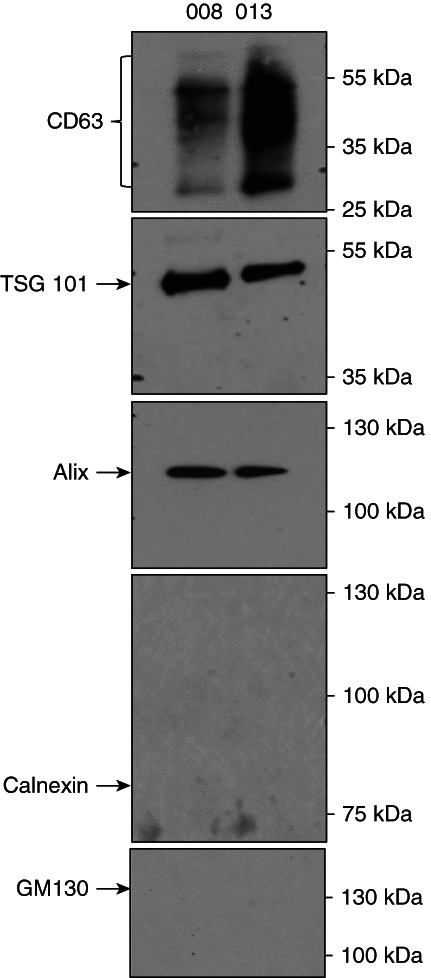
**Representative immunoblots show the expression of exosome markers in urinary EVs.** Urinary EVs were isolated from two samples and characterized by western blotting for the expression of mentioned proteins.

Urine EVs were characterized by western blotting using the exosomal biomarkers CD63, TSG101, and Alix (Figure 2). Endoplasmic reticulum protein Calnexin and Golgi protein GM130 were not present in urinary EVs.

**Figure 3:**
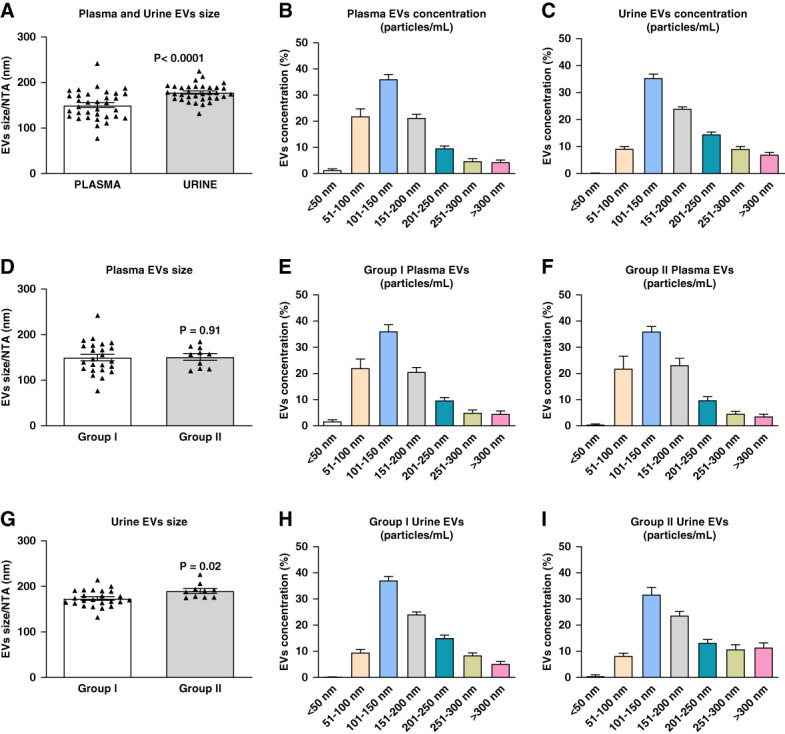
**Comparative scatter graph of EV size and concentrations (particles/mL) in the plasma and urine of patients.** (A-C) Show the data from all patients. (D-I) Illustrate the differences in plasma and urine EV size and concentrations in patients with controlled (Group I) and not controlled (Group II) systolic blood pressure. Bar graphs are Mean ± SE. NTA, nanoparticle tracking analysis.

The size distribution (>50, 51–100, 101–150, 151–200, 201–250, 251–300, and >300 nm) and relative concentrations (particles/ml) of EVs isolated from plasma and urine of controlled and noncontrolled patients are documented in Figure 3. The size of plasma and urine EVs are within the values reported in the literature.^21,23^ Although urine EVs were larger in size (*P* < 0.0001) compared with plasma EVs (Figure 3A), these differences might be accounted for the use of different isolation procedures. Analysis based on hydrodynamic EV size demonstrated a higher concentration of small EVs (<200 nm) in plasma (81%) compared with urine (69%). A higher concentration of medium-to-large EVs (>200 nm) was detected in urine (31%) compared with plasma (19%) (Figure 3, B and C).

Plasma EV size did not differ among patients with or without controlled BP (Figure 3D), whereas urinary EVs were significantly larger in group 2 patients (Figure 3G). No significant differences in plasma EV concentrations were found when compared with corresponding EV sizes between group 1 (<200 nm=80% and >200 nm=20%) and group 2 (<200 nm=82% and >200 nm=18%) patients (Figure 3, E and F). A slightly higher percentage of medium-to-large urinary EVs (group 2 >200 nm=36%) were found when compared with group 1 (>200 nm=29%) patients (Figure 3, H and I).

**Figure 4:**
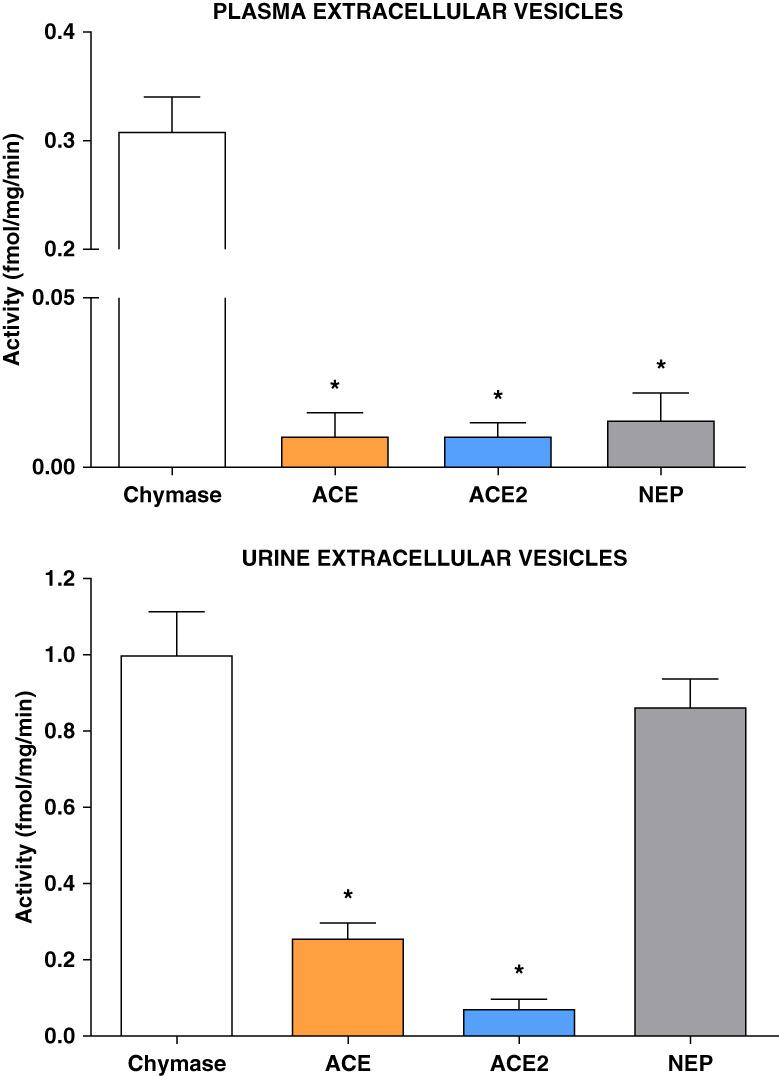
**Enzymatic activity of chymase, ACE, ACE2, and NEP in EVs isolated from the plasma (top graph) and urine (bottom graph) of hypertensive patients.** Values are means±SEM. *P < 0.05 versus chymase activity. *P < 0.05 compared with chymase activity. ACE, angiotensin-converting enzyme; NEP, neprilysin.

Plasma and urine EVs differed significantly in chymase enzymatic activity, averaging 0.31±0.18 and 1.00±0.63 fmol/mg per minute, respectively. Plasma ACE, ACE2, and NEP hydrolytic activities were negligible (Figure 4, top panel). On the other hand, the enzymatic activities of the three enzymes were substantially greater in urine EVs (ACE: 0.26±0.22 fmol/mg per minute, ACE2: 0.07±0.14 fmol/mg per minute, and NEP: 0.87±0.42 fmol/mg per minute) compared with the enzyme activities expressed in plasma EVs (Figure 4, bottom panel). Urinary EVs chymase activity (1.00±0.63 fmol/mg/min) was more than three-fold greater than in plasma EVs, but no different than EVs urinary NEP (0.87±0.42 fmol/mg/min) activity (Figure 4, bottom panel).

**Figure 5:**
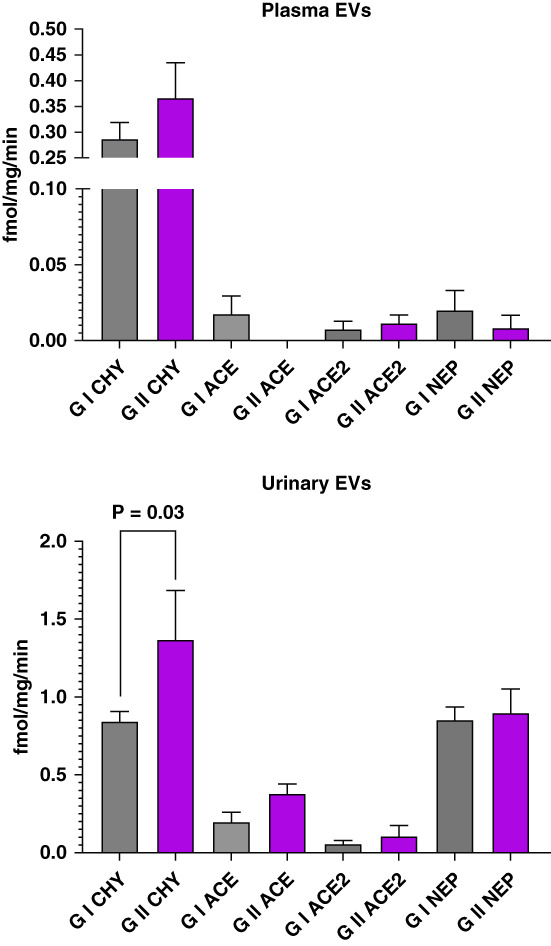
**RAS enzyme activity in plasma and urinary EVs from controlled (G 1) and noncontrolled (G 2) hypertensive patients.** Values are means±SEM. RAS, renin angiotensin system.

There were no differences in the enzymatic activities of chymase, ACE, ACE2, and NEP in EVs isolated from the plasma of controlled and noncontrolled hypertensive patients (Figure 5, top panel). On the other hand, urinary EVs (Figure 5, bottom panel) showed higher values of chymase enzymatic activity in patients with SBP ≥140 mm Hg (group 2: 1.39±1.00 [Mean±SD]; 95% confidence interval, 0.66 to 2.08 fmol/mg per minute) compared with group 1 patients (0.84±0.30 [Mean±SD]; 95% confidence interval, 0.71 to 0.97 fmol/mg per minute).

Potential relationships between the enzymatic activities of plasma and urine angiotensin-generating enzymes found in EVs were explored using the Pearson correlation coefficients.^24^ As documented in Table 2 and the Supplement Material, statistically significant relationships based on an R-value of 0.50 or greater were demonstrated between urine ACE and urine NEP (R=0.71) and urine ACE2 (R=0.53). Urine chymase enzymatic activity correlated with urinary ACE activity (R=0.66) and urinary NEP activity (R=0.59). Plasma chymase enzymatic activity was only moderately correlated (R=0.47) with urinary chymase activity.

**Table 2:** Multiple correlation analysis

Variables	Plasma Chymase (fmol/mg per minute)	Urine Chymase (fmol/mg per minute)	Urine ACE (fmol/mg per minute)	Urine ACE2 (fmol/mg per minute)	Urine NEP (fmol/mg per minute)	Plasma NTA (nm)	Urine NTA (nm)
Plasma chymase, (fmol/mg per minute)	1.00	0.47 (*P* = 0.003)	0.23 (*P* > 0.05)	0.02 (*P* > 0.05)	0.31 (*P* = 0.05)	−0.19 (*P* > 0.05)	0.26 (*P* > 0.05)
Urine chymase, (fmol/mg per minute)		1.00	0.66 (*P* < 0.001)	0.34 (*P* = 0.05)	0.59 (*P* < 0.001)	−0.02 (*P* > 0.05)	0.21 (*P* > 0.05)
Urine ACE, (fmol/mg per minute)			1.00	0.53 (*P* < 0.001)	0.71 (*P* < 0.001)	0.23 (*P* > 0.05)	−0.11 (*P* > 0.05)
Urine ACE2, (fmol/mg per minute)				1.00	0.64 (*P* < 0.001)	0.03 (*P* > 0.05)	−0.01 (*P* > 0.05)
Urine NEP, (fmol/mg per minute)					1.00	0.28 (*P* > 0.05)	0.09 (*P* > 0.05)
Plasma, NTA (nm)						1.00	0.18 (*P* > 0.05)
Urine, NTA (nm)							1.00

The data enclosed in the Table show the multiple linear correlations as the Pearson correlation coefficients (R-value) and *P* values of the enzymatic activities of angiotensin-generating enzymes.

Pearson correlation coefficients. ACE, angiotensin-converting enzyme; NEP, neprilysin; NTA, nanoparticle tracking analysis.

## Discussion

We report significant differences in the enzymatic activity of the main RAS-forming enzymes in EVs isolated from the plasma and urine of hypertensive patients with or without optimal BP control. Highly specific exosomal marker proteins characterized blood and urine EVs.^18,19^ The size of plasma and urine EVs identified in this study is within the values reported for exosomal vesicles.^25^ We further show that urine EVs possess a much higher enzymatic activity for chymase, ACE, ACE2, and NEP. The enriched enzymatic activity of angiotensin-forming enzymes in urine confirms that urine samples reflect the activity of the renal RAS.^26^ Demonstrating a comparatively greater expression of chymase activity over other angiotensin-forming enzymes in plasma suggest a novel mechanism by which chymase contributes to cardiovascular dysregulation. Previous arguments against the *in vivo* importance of chymase as an Ang II forming enzyme are that (*1*) chymase, located intracellularly in mast cells, is unable to interact with Ang I and Ang II^27,28^ and (*2*) the presence of circulating endogenous inhibitors suppresses the activity of chymase once it is released into the interstitial fluid.^29^ Using the technique of cardiac microdialysis, we have previously demonstrated a four-fold increase in ProD-Ala Ang I conversion to Ang II in dogs 3 hours after ischemia reperfusion.^30,31^ We have also demonstrated chymase uptake by rat cardiomyocytes 24 hours after intravenous injection of EVs isolated from the pericardial fluid of patients undergoing cardiac surgery.^32^ This mechanism provides a protected delivery of chymase through the circulation to cells in multiple organs. Here, we take this observation further by demonstrating high chymase activity in EVs free from distortion by *in vitro* tissue homogenization and protected *in vivo* from the serine protease neutralization in the blood and interstitial fluid. Our findings are consistent with a previous demonstration of ACE transfer by EVs to vascular smooth muscle cells in spontaneously hypertensive rats.^33^ Furthermore, the finding that ACE, ACE2, and NEP enzymatic activities were present in only trace amounts in plasma EVs suggests a preferential route through which chymase may contribute to target organ damage.

Tong *et al.*^33^ proposed that exosomes ACE cargoes are indicators of renal dysfunction and structural injury. Their conclusions agree with our demonstration of higher RAS-forming enzyme activities in urine EVs and the evidence of moderate-to-robust correlations of urinary chymase activity with urinary ACE and NEP activities.

It may be argued that circulating serine chymase inhibitors may explain the lower chymase enzymatic activity in blood EVs compared with urine.^34^ Because circulating serpins cannot access the chymase inside the EVs,^35^ the presence of trace ACE, ACE2, and NEP enzymatic activities in circulating EVs suggests a preferential selection of the chymase cargo. The augmented presence of chymase, ACE, ACE2, and NEP activities in urine EVs suggests selective biogenesis of EVs or the extrusion of selective cargo from renal cell membranes.^7^

The potential of these EVs' enzymatic activities as RAS biomarkers in hypertensive individuals is strengthened by demonstrating higher chymase activity in EVs isolated from the blood and urine of medicated, but not controlled, hypertensive patients. We showed higher levels of circulating Ang-(1–12) in primary hypertensive patients' naïve^13^ and not naïve^17^ to antihypertensive therapy. This study expands on those earlier findings by demonstrating higher chymase activity in blood and urine EVs from treated but noncontrolled hypertensive patients. Demonstrating a selective increase in chymase activity in blood and urine EVs suggests that EVs chymase activity acts as a liquid biopsy.^20^

Chymase's role in cardiovascular pathology remains underestimated, partly because its intracellular location excludes access to interstitial or circulating angiotensin I.^28^ The data reported here provide a novel mechanism by which chymase, residing in the protected environment of EVs, transports the enzyme to tissue cells. Our findings contradict the idea that chymase intracellular location voids any functional role as an angiotensin-forming enzyme.^27^ Transferring proteins, enzymes, and lipids locally and systemically to other organs is a fundamental function of EVs.^25^ Our earlier demonstration of human chymase incorporation into rat's cardiomyocytes reveals that exosomes constitute a vehicle for the transport of chymase into organs.^32^

Chymase is abundant in mast cells, contributing to no <25% of the total protein content.^36^ As an ACE-independent source of Ang II formation, mast cell chymase is crucial in the pathogenesis of kidney disease, diabetic nephropathy, and kidney graft rejection.^36,37^ Chymase catalytic activity to generate Ang II from Ang I is twenty-fold higher than ACE,^38^ while chymase, not NEP, is primarily responsible for renal Ang II pathology and its progression to CKD.^39–41^ The potential for these findings to sustain a chymase contribution to the evolution of CKD remains to be unraveled because chymase inhibitors have not shown a beneficial effect on slowing disease progression, the need for KRT or mortality.^42^ Issues with medication dosing and stages at which therapy is initiated need to be further interrogated before chymase inhibitors are excluded in the treatment of chronic kidney failure.^42^

Limitations of this study include the low number of patients incorporated, the underrepresentation of women in the collected data, the observational nature of the study, and a lack of samples from varied ethnic/racial groups. This analysis focuses on the total EVs obtained from plasma and urine, representing the whole body's secretome. Hence, the identity of the cells/organs contributing to their presence remains to be established. Despite these limitations, this study is a considerable advancement toward exploring the contribution of RAS enzymes in EVs to the evolution of primary hypertension.

In summary, enzymatically active chymase activity is the main cargo among major angiotensin-forming enzymes in circulating and urine EVs isolated from hypertensive patients. These data reinforce a chymase contribution as a signaling vector in the molecular processes accounting for cardiovascular pathology and renal dysfunction.

## Supplementary Material

SUPPLEMENTARY MATERIAL

## Data Availability

All data are included in the manuscript and/or supporting information.

## Supplemental Material

Methods

EV Isolation

Immunogold Labeling and Transmission Electron Microscopy (TEM)

Western Blotting

RAS Enzyme Activity Assays

Angiotensin Peptides Radiolabeling and Purification

References
