# Supplementary material for: Chymase Activity in Plasma and Urine Extracellular Vesicles in Primary Hypertension
Source: Kidney360. 2024 Aug 22;5(11):1613–22. doi: 10.34067/KID.0000000000000555 (PMC12282633; doi:10.34067/KID.0000000000000555)
Supplement: SUPPLEMENTARY MATERIAL [file kidney360-5-1613-s002.pdf]

SUPPLEMENT MATERIAL

Chymase Activity in Plasma and Urine Extracellular Vesicles in Primary Hypertension

[1]Sarfaraz Ahmad, [2,3,4]Gagan Deep*, [5]Henry A Punzi, [2]Yixin Su, [2]Sangeeta Singh, [2]Ashish Kumar, [2]Shalini Mishra, [6]Amit K Saha, [1]Kendra N Wright, [1]Jessica L VonCannon, [7,8]Louis J Dell'Italia, [1]Wayne J Meredith, [1]Carlos M Ferrario*

[1]Laboratory of Translational Hypertension, Department of Surgery, Wake Forest School of Medicine, Winston Salem, NC 27157

[2]Department of Internal Medicine, Wake Forest School of Medicine, Winston Salem, NC 27101

[3]J Paul Sticht Center for Healthy Aging and Alzheimer's Prevention, Wake Forest School of Medicine, Winston-Salem, NC 27157

[4]Wake Forest Baptist Comprehensive Cancer Center, Wake Forest School of Medicine, Winston-Salem, NC 27157

[5]Punzi Medical Center Carrollton, TX 75006, and UT Southwestern Medical Center, Dallas, TX 75390

[6]Department of Anesthesiology, Wake Forest School of Medicine, Winston Salem, NC 27157

[7]Division of Cardiovascular Disease, Department of Medicine, The University of Alabama at Birmingham, Birmingham, AL 35487

[8]Department of Veterans Affairs, Birmingham Veterans Affairs Health Care System, Birmingham, AL 35233

Running Head: *Chymase in Human Primary Hypertension*

Table of Content

METHODS 3
EV Isolation 3
Immunogold Labeling and Transmission Electron Microscopy (TEM) 3
Western Blotting 4
RAS Enzyme Activity assays 4
Angiotensin Peptides Radiolabeling and Purification 5
References 5

METHODS

EV Isolation

We isolated plasma EVs by a modified immunoprecipitation method utilizing ExoQuickTM (EXOQ20A-1, System Biosciences, CA) and from urine by ultracentrifugation [1,2]. Briefly, 500 µl of PBS (Ca^{2+} and Mg^{2+} free) was added to 500 µl of plasma, centrifuged at 500 g for 5 min, 2,000 g [room temperature (RT)], and at 10,000 g for 30 min at 4°C to remove larger vesicles. The collected supernatant was mixed with 300 µl of Thromboplastin-D (176065; Fisher Scientific, Hampton) and incubated for 1 hr at RT. After incubation, 700 µL of PBS [Ca^{2+} and Mg^{2+} free and containing a cocktail of protease and phosphate inhibitors (Cat# 1861284, ThermoFisher, MA)] was centrifuged at 1,500 g for 20 min. Then, 252 µL/mL of ExoQuickTM was added to the supernatant, incubated for an additional 1 hr at 4°C, and centrifuged at 1,500 g for 30 min at 4°C. After removal of the supernatant, tubes were centrifuged again at 1500 g for 5 minutes to remove any remaining supernatant or ExoQuick-TC solution. Finally, the pellet was resuspended in 200 µL of 0.1 µm filtered PBS and stored at -80º C until analysis. Similarly, 5.0 mL of urine was centrifuged at 300 g, 2,000 g, and 10,000 g, as described above. The post-10,000 g urine supernatant was passed through a 0.22 µm filter. The filtered supernatant was centrifuged at 100,000 g for 120 min at 4ºC to pellet the EVs. Finally, the pellet was resuspended in 200 µL of 0.1 µm filtered PBS and stored at -80ºC until analysis.

Immunogold Labeling and Transmission Electron Microscopy (TEM)

EVs were visualized using TEM after immunogold labeling [2]. EVs were fixed with 4% paraformaldehyde for 10 min at RT and adsorbed on 200 mesh Copper grids (with carbon-coated formvar film) activated with 100% ethanol. The grids were incubated with EVs at RT for 1 hr and washed 3 times (5 min each) with PBS, followed by 3 times (5 min each) with 50 mM glycine. Grids were blocked with 0.5% BSA in PBS for 30 min at RT and then incubated overnight with rabbit anti-CD63 primary antibody (1:100 dilution, Abcam, MA) at 4°C. The grids were washed 3 times (5 min each) with 0.5% BSA in PBS and incubated at RT with gold-labeled secondary antibody for 2 hr in the dark. The grids were washed thrice

with 0.1% PBST and 0.5% BSA and incubated with 2.5% glutaraldehyde for 5 min. Grids were then washed seven times (5 min each) with 0.1% PBST in 0.5% BSA and incubated with 1% uranyl acetate for 1 min. Finally, the grids were washed with distilled water for 2 min and imaged using TEM (FEI Tecnai Spirit System, Oregon, USA) at 98,000x magnification.

Western Blotting

Twenty-five µg of urine EVs were separated on 12% SDS page gel and transferred to a nitrocellulose membrane. Blots were blocked with 5% non-fat milk for 1 hr and incubated with anti-CD63 (1:500; PA5-92370, Invitrogen, Thermo Fisher Scientific Inc), TSG 101 (1:500; ab83, Abcam), Alix (1:500, ab88388, Abcam, Cambridge, UK), calnexin (1:500, ab22595, Abcam, Cambridge, UK) and GM130 (1:500, NBP2-53420, Novus Biologicals, Centennial, CO) antibody overnight at 4°C. Membranes were washed with 0.1% PBST and incubated with appropriate secondary antibody for 2 hrs at RT. Blots were developed with enhanced chemiluminescence (ECL) reagent (Bio-Rad Inc. CA). In some instances, membranes were stripped and re-probed for another protein of interest.

RAS Enzyme Activity assays

The isolated EVs were lysed by 3-times freeze-thaw cycles in dry ice followed by sonication for 5 sec (Qsonica Sonicators, Newtown, CT, USA). Briefly, the lysed EVs [25-50 µg protein per 200 µL reaction assay buffer in 50 mM Tris-HCl + 150 mM NaCl (pH 8.0) for chymase or 25 mM HEPES + 125 mM NaCl + 10 µM of ZnCl2 (pH 7.4) for ACE, ACE2, and NEP activities] were pre-incubated for 15 min in the absence or presence (50 µM each) of the chymase inhibitor chymostatin, the ACE inhibitor lisinopril, the ACE2 inhibitor MLN-4760, and the NEP inhibitor SCH39370 [3]. After pre-incubation of EVs with different combinations of inhibitor cocktail, a highly purified radiolabeled substrate [1 nmol/L each; ^{125}I-Ang-(1-12) for chymase/ACE/NEP or ^{125}I-Ang II for ACE2] was added to the reaction mixture and incubated overnight at 37°C. At the end of the incubation time, the reaction was stopped by adding an equal volume of ice-cold 1% phosphoric acid, mixed well, and centrifuged 28,000 g for 5 min. The clear supernatants were filtered through a 0.22 µm PVDF membrane syringeless device and samples were injected into the C18 column to separate the substrate and products by HPLC using a linear gradient 10% - 50% mobile phase B (80%

acetonitrile/0.1% phosphoric acid) at 32ºC (flow rate 0.35 mL/min). The solvent system consisted of 0.1% phosphoric acid (mobile phase A) and 80% acetonitrile/0.1% phosphoric acid (mobile phase B). The eluted radiolabeled ^{125}I products were monitored by an in-line flow-through gamma detector (BioScan Inc, Washington, DC), and products were identified by comparison of the retention time of synthetic [^{125}I] standard peptides. Finally, the data were analyzed using Shimadzu LCSolution (Kyoto, Japan) acquisition software. Enzyme activities were calculated based on the amount of parent ^{125}I-Ang substrate hydrolyzed into specific ^{125}I-Ang products by the EVs in the absence and the presence of specific inhibitors.

Angiotensin Peptides Radiolabeling and Purification

Human Ang-(1-12) [DRVYIHPFHLVI] and Ang II [DRVYIHPF] were radiolabeled with ^{125}Iodine-[^{125}I] at the tyrosine 4th residue [Y] using oxidant chloramine-T and purified a C18 column by HPLC as described by us elsewhere [4]. Briefly, 10 µL of 1 mM human Ang-(1–12) or Ang II peptide was added to 20 µL of PBS and 10 µL of 1 mCi of Na[^{125}I] (PerkinElmer, Waltham, MA). The iodination reaction was started by adding 10 µL of chloramine-T solution (15 mg/10 mL MilliQ water) to the mixture for 30 s. The reaction was stopped by adding 50 µL of sodium bisulfate solution (30 mg/10 mL MilliQ water). The iodinated Ang peptide was separated from free Na[^{125}I] by passing the mixture through an activated C18 SPE (solid-phage extraction) column. The SPE eluted iodinated Ang-(1–12) peptide was further purified by HPLC using a linear gradient from 10% to 50% mobile phase B at a flow rate of 0.35 mL/min at 32°C. Products were identified by comparison of retention times of synthetic [^{125}I] standard Ang-(1-12) and Ang II peptides.

References

1. Kumar A, Kim S, Su Y, et al.: Brain cell-derived exosomes in plasma serve as neurodegeneration biomarkers in male cynomolgus monkeys self-administrating oxycodone. EBioMedicine Jan 2021;63:103192. doi:10.1016/j.ebiom.2020.103192

2. Kumar A, Sharma M, Su Y, et al.: Small extracellular vesicles in plasma reveal molecular effects of modified Mediterranean-ketogenic diet in participants with mild cognitive impairment. Brain Commun 2022;4(6):fcac262. doi:10.1093/braincomms/fcac262

3. Trask AJ, Groban L, Westwood BM, et al.: Inhibition of angiotensin-converting enzyme 2 exacerbates cardiac hypertrophy and fibrosis in Ren-2 hypertensive rats. Am J Hypertens Jun 2010;23(6):687-693. doi:10.1038/ajh.2010.51

4. Ahmad S, Punzi HA, Wright KN, Groban L, Ferrario CM: Newly developed radioimmunoassay for Human Angiotensin-(1-12) measurements in plasma and urine. Mol Cell Endocrinol Jun 1 2021;529:111256. doi:10.1016/j.mce.2021.111256
